# Hiding true emotions: micro-expressions in eyes retrospectively concealed by mouth movements

**DOI:** 10.1038/srep22049

**Published:** 2016-02-26

**Authors:** Miho Iwasaki, Yasuki Noguchi

**Affiliations:** 1 Department of Psychology, Graduate School of Humanities, Kobe University, Japan

## Abstract

When we encounter someone we dislike, we may momentarily display a reflexive disgust expression, only to follow-up with a forced smile and greeting. Our daily lives are replete with a mixture of true and fake expressions. Nevertheless, are these fake expressions really effective at hiding our true emotions? Here we show that brief emotional changes in the eyes (micro-expressions, thought to reflect true emotions) can be successfully concealed by follow-up mouth movements (e.g. a smile). In the same manner as backward masking, mouth movements of a face inhibited conscious detection of all types of micro-expressions in that face, even when viewers paid full attention to the eye region. This masking works only in a backward direction, however, because no disrupting effect was observed when the mouth change preceded the eye change. These results provide scientific evidence for everyday behaviours like smiling to dissemble, and further clarify a major reason for the difficulty we face in discriminating genuine from fake emotional expressions.

To what degree is it possible to distinguish genuine from fake facial expressions? Judging the authenticity of facial expressions is indeed an important cognitive skill in various situations, such as criminal (forensic) investigations^1^,^2^, airport security^3^,^4^, business^5^,^6^, health^7^,^8^, education^9^,^10^, and politics^11^. Previous studies have mainly focused on how people discriminate genuine from fake smiles, showing that the major difference lies in muscular activity around eyes^12^. While both genuine and fake smiles share a contraction of the *zygomaticus* major muscle that pulls up the corners of the mouth, only the genuine smile recruits the *orbicularis oculi* muscle around the eyes^13^. A contraction of this muscle causes the distinctive crow’s feet wrinkle that serve as the marker for a genuine smile. Thus it can be said that one’s true emotion (or hidden intention) is represented in the upper half of a smile^14^.

Another cue to judge smile authenticity involves the dynamic features of facial expressions. Because actions of several facial muscles, especially ones strongly related to emotions, cannot be controlled voluntarily^15^, deceptive expressions often involve a leakage of true emotions as a momentary change in facial parts^16^. These leakages of brief duration (1/5th to 1/25th of a second) are called micro-expressions^17^,^18^. Though it is still somewhat controversial^4^, several studies have suggested that micro-expressions may serve as cues for authenticity judgment^19^. In addition to micro-expressions, many static and dynamic cues have been studied for their ability to distinguish genuine from fake smiles, such as left-right asymmetry^20^, the velocity of facial movements^21^,^22^, and rates of eye blinking^23^.

It has also been shown, however, that the rich variety of potential cues may not be fully exploited in actual authenticity judgements. When human observers discriminate genuine from fake expressions of static face images, the mean accuracy is about 70%^24^,^25^,^26^, far from perfect. Performances in real world (dynamic) situations are even worse, slightly better than chance (under 60%)^23^,^27^,^28^,^29^, despite the availability of both static and dynamic cues. This low capacity is not easily improved by learning or experience^1^,^30^,^31^, and it remains unclear why we do not utilize various authenticity cues in actual situations^32^.

Some studies explain the difficulty in authenticity judgements by pointing out the frequent errors people commit when detecting lies, such as the application of cognitive biases (e.g. anchoring heuristics) and neglect of inter-personal (and intra-personal) differences^33^. Other studies have focused on more perceptual or attentional factors that undermine the lie detection process^24^,^34^. They argue that difficulties in discriminating genuine/fake smiles may arise from an omission of or inattention to authenticity cues by human observers, because such cues are subtle and infrequent in most cases. Nevertheless, the validity of this account based on the limitations of perceptual and attentional mechanisms (perceptual-attentional limitation theory) remains a subject of debate^25^,^31^,^32^.

Although many previous studies, especially those focusing on the perceptual-attentional factors mentioned above, have investigated cognitive processes of authenticity judgments using static (single) pictures of faces, facial expressions in real-world situations normally involve dynamic changes in different facial parts at different timings. In the present study, we focus on this dynamic aspect of facial expressions and propose a novel hypothesis to explain the persistent difficulty in authenticity judgements: even when the true emotion of a person is momentarily revealed on his/her face, its conscious detection is inhibited by follow-up movements in other parts of the face, thereby making observers less sensitive to his/her genuine emotions. Since the leakage of true emotions frequently occurs in the upper half of one’s face^14^,^16^, a prime candidate to obscure these emotions would be mouth movements in the adjacent, lower, half. This seems especially likely, considering that the smiling mouth is the most salient feature of all facial expressions^35^. Thus, even when micro-expressions reflecting a true emotion are momentarily revealed by the eyes, the smiling mouth might conceal or neutralize these leakages, resulting in poor performance in authenticity judgements in the real world.

Based on this idea, we examined whether the detection of micro-expressions in the eye region is actually prevented by mouth movements. As shown in Fig. 1a, we made video-type (dynamic) facial stimuli in which a brief change in the eye region was accompanied by a gradual change in the mouth region. These dynamic changes were created via morphing still images in a facial expression database (see Methods). We set four conditions in which an eye change (micro-expressions) and a mouth change (from neutral into a smile) were independently manipulated. Participants judged presence/absence of the eye change ignoring the mouth change. (Note that although faces in Figs 1, 2, 3, 4 are depicted using line drawings, for copyright reasons, all experiments were actually performed using photographs of real faces taken from a database developed for academic researchers; see Methods). If the mouth change disrupts the detection of the eye change, task accuracy might be lowered in a condition where both the eyes and mouth change simultaneously (condition 4 in Fig. 1a) compared to a condition involving only an eye change (condition 2).

## Results

### Decreased sensitivity to micro-expressions in the presence of mouth movements

**Figure 1:**
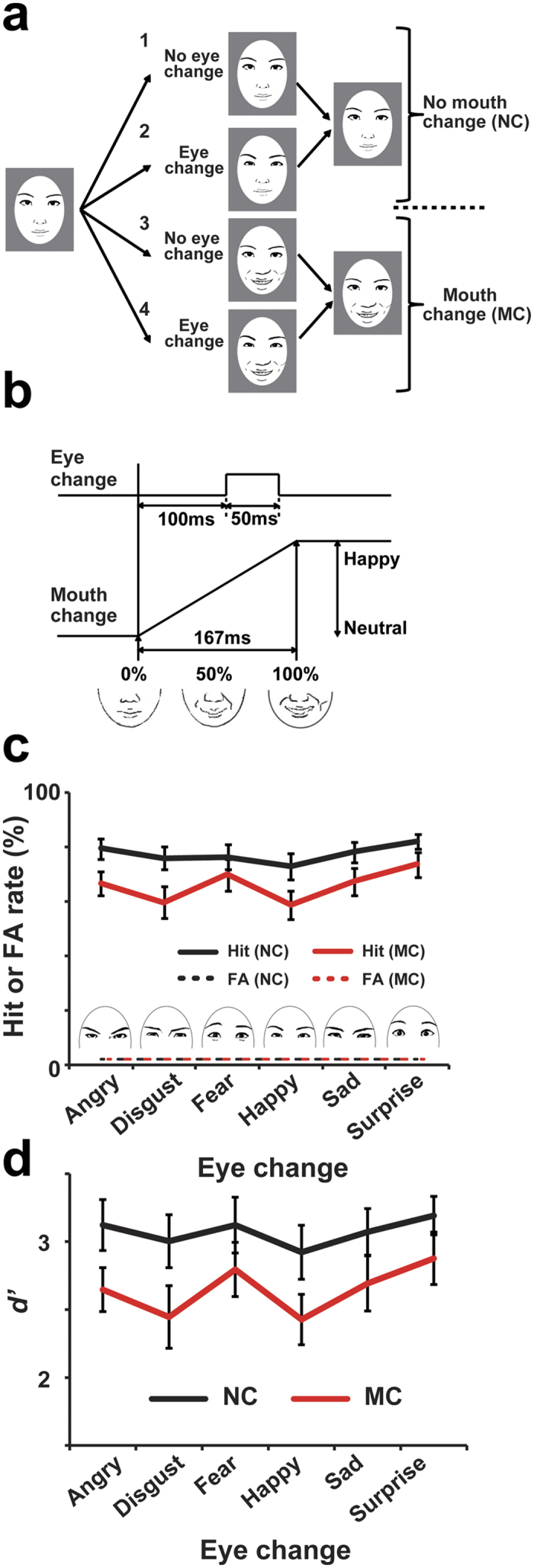
Experiment 1 (**a**) Four basic conditions. We independently manipulated changes in eye (upper) and mouth (lower) regions of a face across trials. In this figure, an eye region briefly changes into a happy expression in conditions 2 and 4 (micro-expression), while conditions 3 and 4 involve movements of a mouth. Participants judged presence/absence of the eye change ignoring the mouth change (eye detection task). (**b**) Relative timing of the eye and mouth changes in MC (mouth change) trials. The eye change started at 100 ms after onset of the mouth change. (**c**) Hit and false alarm (FA) rates in the eye detection task for six types of micro-expressions. The hit rates were decreased for all micro-expressions in the MC condition (red solid lines) compared to the NC condition (black solid lines), though FA rates were comparable between conditions (dotted lines). (**d**) The *d’* obtained from the hit and FA rates. A two-way ANOVA of the six types of eye changes × mouth change (MC/NC) indicates a significant main effect of mouth change (MC < NC). There was no main effect of the eye changes or an interaction. In these and subsequent figures, all error bars denote standard errors (SEs) across participants. Note that, although faces in Figs 1, 2, 3, 4 are shown in line drawings for copyright reasons, all experiments were actually performed using photographs of real faces taken from a database developed for academic researches (ATR DB99). See our previous study for sample images from this database^52^.

Figure 1c shows hit (solid lines) and false-alarm (FA, dotted lines) rates for the detection task of micro-expressions of the eyes. Trials of female and male faces were pooled. For all six types of micro-expressions, we found a decrease in the hit rates when an eye change was accompanied by a mouth change (MC, mouth change condition, red lines) compared to when not (NC, no mouth change, black lines), although the FA rates were comparable between those two conditions. The *d’*, obtained from the hit and FA rates is given in Fig. 1d. A two-way ANOVA on the six types of eye changes and the presence/absence of mouth change yielded a significant main effect of mouth change (MC < NC, *F*(1, 18) = 12.62, *p* = 0.002, *η*^2^ = 0.41). No main effect of the eye changes (*F*(2.54, 45.68) = 1.69, *p* = 0.189, *η*^2^ = 0.09) or interaction (*F*(5, 90) = 0.804, *p* = 0.55, *η*^2^ = 0.04) was observed. These results indicate that a dynamic change from a neutral to a smiling mouth disrupted the detection of all types of micro-expressions tested in the present study.

### The critical role of perceptual continuity between upper and lower facial parts

**Figure 2:**
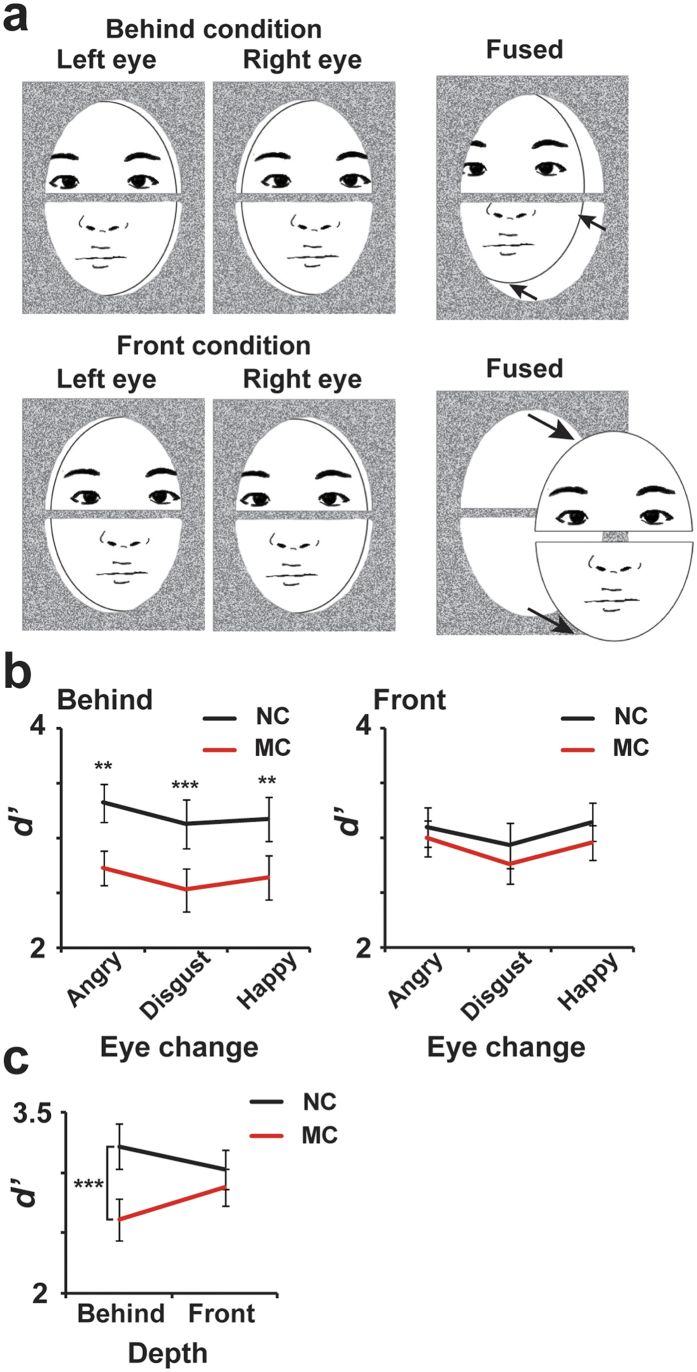
Experiment 2. (**a**) Images presented to left and right eyes of a participant. Stereoscopic images when fused between two eyes are shown in the rightmost panels. In the behind condition (upper panels), upper and lower parts of a face were perceived to be stereoscopically behind a random-dot pattern, which enabled the participant to see a coherent image of a face behind an occluding pattern (amodal completion). This integrity (perceptual continuity) between the two parts was disconnected in the front condition (lower panels) where they were presented stereoscopically in front of the random-dot pattern. (**b**) The *d’* in the eye detection task in the behind and front conditions. For all three types of micro-expression in eyes (anger, disgust, and happiness), a significant reduction in *d’* by the mouth change was selectively observed in the behind condition. (**c**) The *d’* combined across the three eye emotions. An ANOVA indicated a significant interaction between depth (behind/front) and mouth change (MC/NC). Post-hoc comparisons indicated a significant reduction of *d’* in the behind but not in the front condition. **p < 0.01. ***p < 0.001.

It remained to be elucidated in Experiment 1 why the task-irrelevant movements of the mouth prevented a conscious detection of micro-expressions in eyes. One possibility was that mouth movement distracted attention to the eye region. Because a smiling mouth is the most distinct facial feature of all facial expressions^35^, the mouth change in Experiment 1 might have strongly drawn participants’ attention to a lower part of the face, causing an insensitivity to micro-expressions in the eyes (inattentional blindness). Another possibility involves the holistic (or object-based) processing of faces. In the high-level visual cortex along the ventral pathway, multiple features of visual stimuli are integrated into a representation of an object^36^. A face (especially an upright face) is a typical example of this object-based processing^37^. Many studies further emphasize the role of holistic face processing^38^ whereby facial parts (e.g. the eyes, nose, and mouth) are processed interactively rather than independently. It might be possible, therefore, that the mouth movements in Experiment 1 somehow affected the perception of a distant eye region via an object-based linkage between the lower and upper halves of the face, resulting in a decrease in *d’* in the MC versus the NC conditions. In order to clarify these two possibilities, in Experiment 2 we employed a psychophysical paradigm called amodal completion^39^. Specifically, we created two conditions (behind and front) where an identical face image was perceived to be in different depth positions (3D layers) by manipulating a disparity in binocular images. In the behind condition (upper panels in Fig. 2a), we presented upper and lower parts of a face so that they appeared stereoscopically behind an area of visual noise (random-dot pattern). This facilitated a perceptual integration of the two parts, enabling the participants to see a coherent image of a face behind the occluding dot pattern (amodal completion). Meanwhile, the integrity (perceptual continuity) between the two parts was broken in the front condition (lower panels in Fig. 2a) where they were presented stereoscopically in front of the visual noise and thus appeared to be disjoint parts of a face. If a reduction in *d’* by mouth movements (Fig. 1d) was caused by holistic facial processing, the disconnection of perceptual continuity in the front condition would hamper an interaction between the eye and mouth regions, resulting in no effect of mouth movements on the detection of eye changes. In contrast, if the reduction in *d’* (Fig. 1d) reflected the distraction of attention, it would be observed equally in both the behind and front conditions because the two conditions shared common patterns of 2D mouth movements that would attract attention of viewers.

The results of Experiment 2 (*d’* in detecting the micro-expressions) are shown in Fig. 2b,c. Here, we focused on three emotions (happiness, anger, and disgust) out of the six tested in Experiment 1 because these three showed greater reductions in *d’* by mouth movements (Fig. 1d) compared to the other three emotions (fear, sadness, and surprise). We conducted a three-way ANOVA on the three types of micro-expressions, the presence (MC) or absence (NC) of mouth change, and the depth of stimuli (behind or front condition.) There were main effects of mouth change (*F*(1, 16) = 9.62, *p* = 0.007, *η*^2^ = 0.38) and eye change (*F*(2, 32) = 3.41, *p* = 0.045, *η*^2^ = 0.18) as well as a significant interaction between depth and mouth change (*F*(1, 16) = 7.08, *p* = 0.017, *η*^2^ = 0.31). No other main effect or interaction was observed (*F* < 0.63, *p* > 0.52, *η*^2^ < 0.38). Post-hoc comparisons on the depth × mouth change interaction indicated a significant reduction of *d’* in the behind (*p* = 0.0002, *d* = 0.82) but not in the front (*p* = 0.16, *d* = 0.24) conditions. These results show that a perceptual continuity between the upper and lower halves of the face play a critical role in the decrease in *d’* observed in Experiment 1 (Fig. 1d), suggesting that reduced sensitivity to micro-expressions resulted from an object-based facial processing, rather than from distraction of attention by mouth movements.

### Backward masking of eye changes by subsequent mouth changes

In Experiments 1 and 2, we showed that a detection of micro-expressions in the eye region was prevented by simultaneously presenting a mouth change. In Experiment 3, we further investigated whether this prevention would also be observed when eye and mouth changes occurred in separate time windows. Specifically, we tested a condition where mouth movement preceded eye change (Exp. 3a) and a condition where mouth movement followed eye change (Exp. 3b). If reduced sensitivity to micro-expressions (Figs 1d and 2b) resulted from a temporal interaction between two visual events (e.g., target and mask stimuli in visual masking), mouth change would affect perception of eye changes even when there was no temporal overlap between the two.

**Figure 3:**
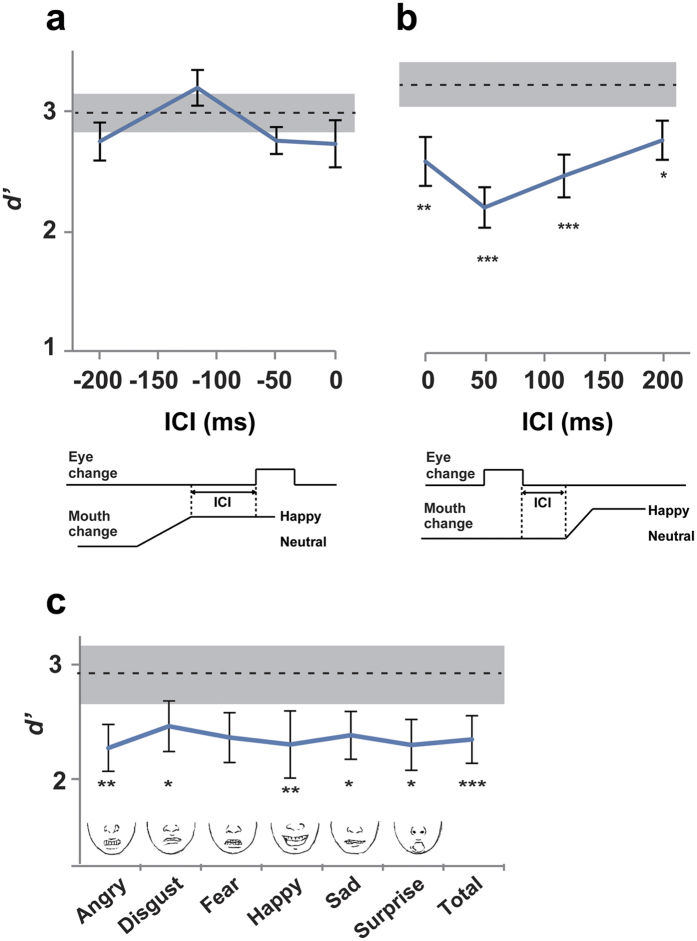
Experiment 3 and Experiment 4. (**a**) Experiment 3a (mouth change first). An inter-change interval from the offset of mouth change to the onset of eye change was randomly varied across trials from -200 to 0 ms. The *d*’ at four ICIs were not significantly different from the *d’* in the control (NC) condition (dotted line). A background shading denotes SEs across participants in the NC condition. (**b**) Experiment 3b (eye change first). The detection of eye change was most severely impaired when the mouth change was delayed compared to the eye change by 50 ms. (**c**) Experiment 4. The *d’* at each of six MC conditions and across the total of all MC conditions was decreased from that of the NC condition (dotted line). *p < 0.05. **p < 0.01. ***p < 0.001.

In Experiment 3a, an inter-change interval (ICI) from the offset of mouth change to the onset of eye change was randomly varied across trials either at 200, 117, 50, or 0 ms. The results (*d’* averaged across the three types of micro-expressions) are shown in Fig. 3a. First, a one-way ANOVA of *d’* across the five conditions indicated no significant main effect (*F*(4, 36) = 2.524, *p* = 0.058, *η*^2^ = 0.219). Mouth change thus did not affect a perception of eye change when it preceded eye change. Figure 3b shows the results of Experiment 3b. This time, a one-way ANOVA of *d’* across the five conditions revealed a significant main effect (*F*(4, 36) = 15.174, *p* < 0.001, *η*^2^ = 0.628). We evaluated the effect of mouth change on the detection of eye changes by comparing *d’* between the NC (no mouth change) condition and each of four MC conditions with different ICIs. Because we repeated *t*-tests four times, *p*-values were corrected according to the Bonferroni method. Significant differences were found in NC vs. ICI 0 ms (*t*(9) = 4.86, corrected *p* = 0.003, *d* = 1.01), NC vs. ICI 50 ms (*t*(9) = 7.52, corrected *p* < 0.001, *d* = 1.80), NC vs. ICI 117 ms (*t*(9) = 10.57, corrected *p* < 0.001, *d* = 1.29), and NC vs. ICI 200 ms (*t*(9) = 3.12, corrected *p* = 0.049, *d* = 0.81). These asymmetric results between Experiment 3a and 3b were not caused by a difference in task difficulty. A comparison of *d’* in the NC (control) condition between Experiments 3a and Experiment 3b indicated no significant difference (*t*(18) = -0.95, *p* = 0.35, *d* = 0.42), suggesting that task difficulty was balanced between the two experiments.

Taken together, our results indicate that micro-expression perception is not disrupted by a mouth change preceding an eye change (Exp. 3a), but is disrupted when the mouth change occurs during (Exps. 1 and 2) and after (within 200 ms, Exp. 3b) an eye change. The magnitude of this disruption reaches its maximum value when the mouth change occurs at 50 ms after the eye change, resembling the U-shaped curve of task accuracy (as a function of target-mask asynchrony) characteristic of the backward masking paradigm.

### The masking effect caused by various types of mouth changes

The previous experiments used a smiling mouth as the facial expression displayed in the lower half of stimuli presentations. In Experiment 4, we explored whether a similar masking effect could be induced by various types of mouth changes other than a smile. Trials with six types of mouth changes (MC trials; happiness, anger, disgust, sadness, fear, and surprise) were randomly intermixed with the NC (no mouth change) trials in each session of Experiment 4. We evaluated the effect of mouth changes by comparing the *d’*, sensitivity to three types of micro-expressions (happiness, anger, disgust), between the NC and six MC conditions (Fig. 3c). A one-way ANOVA of *d’* across the seven conditions indicated a significant main effect (*F*(6, 66) = 4.453, *p* = 0.001, *η*^2^ = 0.29). Planned comparisons of the NC condition with each of the MC conditions (corrected for multiple comparisons by the Bonferroni method) showed significant reductions of *d’* for anger (*t*(11) = 5.10, corrected *p* = 0.002, *d* = 0.80), disgust (*t*(11) = 3.63, *p* = 0.023, *d* = 0.54), happiness (*t*(11) = 5.09, corrected *p* = 0.002, *d* = 0.64), sadness (*t*(11) = 3.86, *p* = 0.015, *d* = 0.66), and surprise (*t*(11) = 3.97, *p* = 0.013, *d* = 0.74), but not for fear (*t*(11) = 2.93, corrected *p* = 0.081, *d* = 0.67).

## Discussion

Although perception of facial expressions has been traditionally studied using static images, recent studies emphasize the importance of dynamic aspects of expressive changes^40^,^41^,^42^,^43^,^44^. The present study provides an example of a dynamic interaction between the upper and lower halves of the face that can hamper our authenticity judgements in the real world. Although the numbers of participants in our experiments were relatively small (10–19), these experiments evince converging results that a dynamic change in the mouth region inhibits the detection of a brief change in the eye region. This consistency across experiments, along with the statistical power (*β* of > 0.6) of our analyses, highlights the robustness of our results.

**Figure 4:**
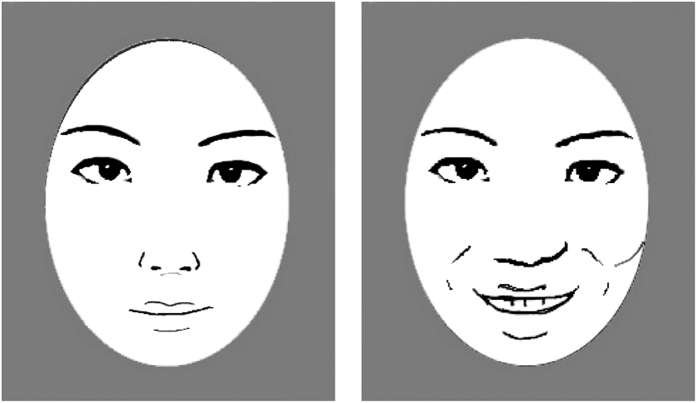
Perceptual bias induced by a smile^45^,^46^. Although the left and right faces share the same image of eyes with a neutral expression, the eyes on the right face are perceived to be happier than the left ones.

Why do mouth movements affect the perception of changes in a distant eye region? One key factor could be a perceptual bias caused by the smiling mouth^45^,^46^. It is known that a picture of a smiling mouth biases perceived expression in other facial parts (e.g., eyes) toward happiness, even when an actual eye image is not happy (Fig. 4). A dynamic display in the present study would induce this bias (a “projection” of happiness from the mouth to the eyes) synchronized with mouth movements. It is possible that the bias projected from a smiling mouth interfered with physical motion signals in the eyes (micro-expressions), resulting in a reduction of sensitivity (*d’*) to micro-expressions (although our present results were not sufficient to provide direct evidence for this possibility). Interestingly, the reduced sensitivity to micro-expression was not observed when we disconnected perceptual continuity between upper and lower facial parts (front condition in Exp. 2). Therefore the failure to detect a change in the eye region was induced only when those two parts were recognized as a unified object in the brain. In the high level visual areas along the ventral pathway, multiple features of visual stimuli are integrated into a representation of an object^36^. An important role of perceptual continuity shown in Experiment 2 suggests that the masking effect in the present study emerges from object-based processing in high level visual regions.

One should note that the present masking effect is different from emotional neutralization or mixture between positive and negative expressions^47^,^48^. For example, participants in Calvo *et al.* (2012) categorized various expressions into “happy” or “unhappy”. Although the percentages of “happy” response to negative expression images (angry, disgusted, sad and fearful) were less than 2%, these responses were elevated to more than 30% for chimera faces where negative eye images were combined with a smiling mouth, suggesting that negative emotions in the eyes are neutralized by a positive emotion in the mouth. On the other hand, our present effect represents a perceptual (as opposed to emotional) masking, a more radical type of prevention in which emotional signals in the eye region are made undetectable to observers. Contrary to the concept of neutralization, our smiling mouth equally disrupted the detection of eye changes (Exp. 1) regardless of whether the eye change was emotionally positive (happy) or negative (angry, disgusted, fearful, or sad).

The fact that the masking effect was maximized when a mouth change followed an eye change (Exp. 3) has some implications for facial communication in the real world. Since the facial leakage of a true emotion (including micro-expressions) cannot be controlled voluntarily^15^,^16^, it is probable that such automatic facial changes arise quickly and reflectively in response to triggering events, followed by slow voluntary facial movements that act to hide them. The backward nature of the present effect (Exp. 3) suggests that these mouth changes after the reflective facial responses actually happen in the most effective time periods to hinder true emotions. Thus the follow-up mouth movements might reflect a highly-adaptive strategy utilizing a neural mechanism that inhibits conscious perception of a preceding event.

In conclusion, the present study demonstrated a new type of dynamic masking between the upper and lower halves of the face. This masking effect was so strong that an insensitivity to micro-expressions unavoidably occurred even when full attention was paid to the eye region. Furthermore, this masking can be induced by a wide range of mouth movements (Exp. 4), suggesting that it happens routinely in everyday face-to-face conversations. Beyond their scientific implications, we hope our findings will provide practical insight for various fields concerned with decoding the truth of emotions, including but not limited to law enforcement^2^ and international security^3^,^4^.

## Methods

### Participants

We conducted five (Exps.1, 2, 3a, 3b and 4) experiments in the present study. Numbers of participants were 19, 17, 10, 10, and 12 respectively. All participants had normal or corrected-to-normal vision. We tested participants’ autism tendency using Autism-Spectrum Quotient^49^ and confirmed that the scores of all participants were within a normal range (mean ± SD: 21 ± 7.1, range: 10–37). Informed consent was received from each participant after the nature of the study had been explained. All experiments were carried out in accordance with guidelines and regulations approved by the ethics committee of Kobe University, Japan.

### Stimuli

All visual stimuli were generated using the MATLAB Psychophysics Toolbox^50^,^51^ and presented on a CRT monitor (resolution: 1024 × 768 pixels, refresh rate: 60 Hz). The participants viewed dynamic movies of human faces whose expressions in the upper and lower halves were independently changed. Image materials for these movies were taken from a facial expression image database (ATR DB99, ATR-Promotions, Kyoto, Japan). We selected photographs of two persons (female and male, Figs 1a and 2a) showing typical expressions of six basic emotions (happiness, anger, disgust, sadness, fear, and surprise). The mean luminance of those images was 28.8 cd/m^2^ (female) and 28.9 cd/m^2^ (male). We cut a central region of each image into an oval shape (6.875 × 8.75 deg.), discarding non-facial areas (e.g., hair, neck) in the periphery. To make dynamic display of expression changes, these images were morphed into a set of pictures that ranged from neutral (0%) to one of six basic emotions (e.g. 100% happiness), using an image-processing software package which is available online (Win Morph, http://www.debugmode.com/winmorph/). Movements from neutral to a smiling mouth (duration: 167 ms), for example, were shown by sequentially presenting 10 images from 0 (neutral) to 100% (happy) at a rate of 16.7 ms per image.

### Experiment 1

Each trial in Experiment 1 started with a fixation of 500 ms, followed by a neutral face of either a female or male (500–900 ms, randomly varied across trials). There were four types of trials depending on a presence/absence of changes in the upper (eye region) and lower (mouth region) halves of the face. The change in eye region was a micro-expression in which a neutral image was briefly (50 ms) replaced by one of six emotions. The magnitude of the emotional change was determined for each participant so that (s)he could detect that change with an accuracy of 70–80%. The change in the lower region, on the other hand, involved a gradual shift from neutral to a smiling mouth over 167 ms (see above). Note that, while the micro-expression in eye region disappeared within 50 ms, the smiling mouth was continuously presented till the end of each trial. When a trial underwent both types of changes simultaneously, the eye change began at 100 ms after an onset of the mouth change (Fig. 1b). Participants were instructed to judge the presence/absence of the micro-expression, ignoring the mouth region. They pressed one button when they detected any types of eye change and pressed another when they did not. No time limitation was imposed. We monitored eye movements of participants at 500 Hz using the EyeLink CL system (SR research, Ontario, Canada).

Participants performed six experimental sessions of 96 trials (48 for the female and 48 for the male faces). Each session contained equal numbers of four types of trials (eye change/no change × mouth change/no change) randomly intermixed. The order of the six types of micro-expressions was also randomized across trials.

We analyzed the data by calculating hit and false-alarm (FA) rates for a detection of eye changes. Those rates were then converted into a sensitivity index (*d’*) in a manner accordant with signal detection theory. The effect of mouth movements on the detection of micro-expressions was quantified by comparing *d’* between the mouth change trials (MC) and no mouth change trials (NC).

### Experiment 2

The basic procedures in Experiment 2 were identical to Experiment 1, except for several modifications listed below. First, we focused on three types of eye changes (happiness, anger, and disgust) out of the six emotions tested in Experiment 1. These three showed greater reductions in *d’* by mouth movements (Fig. 1d) compared to the other three emotions (fear, sadness, and surprise). Second, 3D positions of facial images were varied across conditions by manipulating a disparity of binocular images. A rectangular area (delineated by white lines, 6.875° × 8.75°) on a left half of the screen contained stimuli (facial images on a random-dot plane) for the left eye of a participant, whereas another area on the right half comprised those for the right eye. Stimuli at these two locations were fused with a mirror stereoscope. The facial images and their movements were perceived to be behind the random-dot plane in one condition (upper panels in Fig. 2a, behind conditions) but to be in front of the plane in another condition (lower panels in Fig. 2a, front condition). Note that these two conditions were balanced in terms of visual inputs, except that the images presented to one eye in the behind condition were given to the other eye in the front condition, etc. Disparities between binocular images were 0.156 deg. for the front and −0.156 deg. for the behind conditions. Trials in the behind and front conditions were conducted in separate experimental sessions with their orders counterbalanced across the participants. As with Experiment 1, participants judged whether the upper half of the face underwent any changes or not (detection of micro-expressions). Each experimental session comprised 96 trials, 48 with eye changes and 48 without eye changes. The 48 eye-change trials consisted of equal numbers of 12 conditions produced by a combination of three types of micro-expressions (happiness/anger/disgust) with a mouth change (present/absent) and face identities (female/male). The 48 no-eye-change trials consisted of four conditions crossing the mouth change with face identities. Participants performed six sessions (three for the behind and three for the front conditions) in which all kinds of trials were randomly intermixed.

### Experiment 3

Although the fundamental procedures were the same as Experiments 1, several modifications were made. First, as in Experiment 2, we focused on three types of eye changes (happiness, anger, and disgust) that had shown greatest reduction in *d’* in the presence of mouth movement (Fig. 1d). Second, while the micro-expression (eye change) was always synchronized with the mouth change in Experiments 1 and 2, we presented those changes in different time windows. In Experiment 3a (mouth change then eye change), an inter-change interval (ICI) from the offset of a mouth change to an onset of an eye change was randomly chosen from 200, 117, 50, and 0 ms. The ICI in Experiment 3b (from the offset of an eye change to the onset of a mouth change) was also set at either 0, 50, 117 or 200 ms. Participants performed five experimental sessions of 120 trials, 60 with eye changes and 60 without eye changes. The 60 eye-change trials consisted of equal numbers of 30 conditions produced by a combination of three types of micro-expressions (happiness/anger/disgust) with five types of mouth changes (four MC conditions with different ICIs from 0 to 200 ms plus the NC condition as a control) and face identities (female/male). The 60 no-eye-change trials consisted of 10 conditions crossing the mouth change with face identities. All kinds of trials were randomly intermixed.

### Experiment 4

The procedure was the same as in Experiment 1 above with some exceptions. First, we employed six types of mouth changes (happiness, anger, disgust, sadness, fear, and surprise) in Experiment 4, although only a single mouth change into smile (happiness) had been used in prior experiments. The stimuli involved a gradual shift of emotional magnitude from 0% (neutral) to 100% (typical mouth image) in each expression over 167 ms. Second, in order to maximize the masking effect, the mouth change began at 50 ms after the offset of the eye change. As with Experiments 2 and 3, we tested three types of eye changes (happiness, anger, and disgust). Participants underwent 588 trials, 294 with eye changes and 294 without eye changes. The 294 eye change trials consisted of equal numbers of 42 conditions produced by a combination of three types of micro-expressions in eyes (anger/disgust/happiness) with seven types of mouth changes (no-change/anger/disgust/fear/happiness/sadness/surprise) and face identities (female/male). Participants performed six experimental sessions of 98 trials where trials in all conditions were randomly intermixed.

## Additional Information

**How to cite this article**: Iwasaki, M. and Noguchi, Y. Hiding true emotions: micro-expressions in eyes retrospectively concealed by mouth movements. *Sci. Rep.* **6**, 22049; doi: 10.1038/srep22049 (2016).
